# Integrating Sensory Evaluation, Electronic Nose, and Metabolomics to Characterize Aroma in Peach and Nectarine Varieties

**DOI:** 10.3390/foods14173087

**Published:** 2025-09-02

**Authors:** Meng Sun, Julin Ma, Zhixiang Cai, Juan Yan, Ruijuan Ma, Mingliang Yu, Yinfeng Xie, Zhijun Shen

**Affiliations:** 1 College of Horticulture, Jinling Institute of Technology, Nanjing 210038, China; sm183495665@163.com; 2 Institute of Pomology, Jiangsu Academy of Agricultural Sciences, Nanjing 210014, China; 3 College of Life Sciences, Nanjing Forestry University, Nanjing 210037, China

**Keywords:** peach, sensory evaluation, electronic nose, metabolomics

## Abstract

This study investigates the aroma differences among various peach and nectarine varieties by sensory evaluation, electronic nose (E-nose) analysis, and metabolomics. Peach is a significant fruit crop in China, and identifying unique fragrances is essential for germplasm selection and cultivar improvement. Six peach and nectarine varieties were collected from the National Peach Germplasm Repository in Nanjing, China. Sensory evaluation revealed significant differences in aroma and taste, with ”Zi Jin Hong 3” and “Bai Mi Pan Tao” showing high scores for aroma, sweetness, and overall sensory quality, while “Tachibanawase” had the lowest overall impression score. E-nose analysis showed distinct response values among varieties, with sensors W1S, W1W, and W5S exhibiting the highest sensitivity. GC-MS identified 446 metabolites, including esters and terpenes. PCA and OPLS-DA differentiated metabolite profiles among varieties, revealing significant differences in metabolite expression. The integration of these techniques provides a comprehensive understanding of aroma differences, highlighting the potential for identifying unique germplasms for breeding high-quality cultivars with charming flavor, and offering a theoretical foundation for raw material selection and process optimization in the deep-processing industry of peach fruits in future research.

## 1. Introduction

Peach (*Prunus persica* (L.) Batsch) and nectarine (*Prunus persica* var. nectarina) rank among China’s significant fruits and are cultivated across the entire country. They both belong to *Prunus persica* in the Rosaceae family—essentially, they are different phenotypes of the same species. Their most prominent distinction is the presence or absence of skin pubescence. Peaches typically have a skin covered with fine, dense hair (referred to as “skin pubescence”), while nectarines have a smooth, hairless skin [1]. They share high similarity in key quality-related dimensions like chemical composition and aroma characteristics. Their flesh is delicate, juicy, aromatic and nutritious. It is widely beloved and holds significant economic and ecological value [2]. The yield and quality of peaches and nectarines are crucial economic factors. Aroma is a vital component of peach flavor and a major quality trait influencing consumer preference [3,4]. Given the abundant germplasm resources and extensive varietal diversity in peaches, which results in distinct aromatic profiles, the identification of cultivars with unique fragrance is crucial for germplasm evaluation and genetic improvement [5,6].

In our prior research, we conducted sensory evaluations on 356 peach germplasm resources, with a focus on aroma, taste (sweetness, sourness, astringency and bitterness), aftertaste and overall acceptability [7]. Screening peach germplasms that exhibit strong sweetness and a suitable characteristic aroma is vital for improving peach quality. Building on the sensory assessment results, 231 representative germplasm resources were chosen to carry out systematic evaluation and differentiation of aroma types, laying the groundwork for electronic nose analysis and providing a basis for peach production and quality enhancement. Hierarchical clustering divided the 231 peach germplasms into six groups using data from 10 E-nose sensors [7,8]. One representative germplasm from each group was chosen for metabolomic analysis. Based on combined sensory and E-nose results, the selected samples were “Xuan Cheng Tian Tao”, “Jin Xia Zao You Pan”, “Zi Jin Hong 3”, “Tachibanawase”, “Mangold” and “Bai Mi Pan Tao”.

Metabolomics is widely applied in food analysis. It enhances our understanding of intracellular metabolite dynamics and is considered closer to phenotypic expression than other “omics” approaches [9,10]. Metabolites, serving as biomarkers for plant phenotypes, reflect physiological and biochemical traits and reveal gene expression, signaling and metabolic pathways. Gas chromatography–mass spectrometry (GC-MS), offering high resolution and sensitivity but requiring sample derivatization, is primarily used for volatile compound detection and has been extensively applied in fruit flavor analysis [10,11,12,13].

Beyond peaches, quality improvement of other fruits and vegetables (such as apples, grapes and tomatoes) also relies on accurate analysis of characteristic flavor compounds [14]. In current research, single detection techniques are insufficient to comprehensively characterize quality differences among fruit. In contrast, a multi-dimensional approach integrating sensory evaluation, instrumental analysis and omics technologies can provide a universal framework for germplasm screening and cultivar improvement across different fruits. The analytical system established in this study, using peaches as a case study, with its core methodology, can be extended to other agricultural products, offering a standardized tool for quality enhancement in agriculture. Additionally, the findings provide resources and data support for breeding and the peach processing industry.

## 2. Materials and Methods

### 2.1. Plant Materials

Peaches and nectarines (see Table 1) were sourced from the National Peach Germplasm Repository located in Nanjing, China (geographical coordinates: 32°20′ N, 118°52′ E; altitude: 11 m) in June and July 2022. For each cultivar, two trees were selected as representatives. The test varieties consisted of 5-year-old mature trees trained to a Y-shaped structure, with a row spacing ranging from 3 to 5 m, and they were managed under conventional cultivation practices as described by Zhao et al. [8].

At the stage of maturity—defined as the complete disappearance of green coloration on the fruit base—50 fruits (peaches or nectarines) were randomly sampled from the two trees of each variety. All harvests were conducted before 9:00 a.m. Each fruit was halved and pitted: one half was transported to the sensory evaluation room for assessment by a trained panel, while the other half was immediately frozen and stored at −80 °C for subsequent electronic nose (E-nose) analysis and GC-MS measurements.

### 2.2. Sensory Analysis

Sensory panel training and subsequent sensory evaluations were conducted using the quantitative descriptive analysis method, following the guidelines outlined in Sensory Analysis—Methodology—Paired Comparison Test (ISO 5495:2005, International Organization for Standardization: Geneva, Switzerland, 2005) [15]. To enable panel members to recognize qualitative characteristics, various peach and nectarine samples were provided for training.

Ten candidates (five females and five males, consisting of postgraduate students and researchers from JAAS) were pre-screened based on their availability, health status and general dietary habits. They volunteered for the project, and all provided informed consent prior to participation. The fruits used in the study were sourced from the National Peach Germplasm Repository in Nanjing, China, and were confirmed safe for sensory research. All experimental procedures involving human volunteers adhered to the Declaration of Helsinki. Additionally, the selected panelists demonstrated the ability to discriminate between products based on basic taste thresholds and to articulate their sensory perceptions. Through brainstorming and round-table discussions, the panel collectively developed descriptions and definitions for key attributes of peaches and nectarines, including flavour, palate, persistence and overall impression.

Subsequently, a scoring sheet was designed to assess these attributes. Flavour was evaluated across seven dimensions: fruity, floral, earthy, woody, caramel, nutty and herbaceous/vegetative. Palate (encompassing aroma, sweetness, sourness, bitterness and astringency), persistence, and overall impression were rated using a categorical scale from 0 to 10, with the following criteria: 0 = no sensation; 0.1–1.9 = negligible sensation; 2.0–4.9 = weak; 5.0–7.9 = moderate; and 8.0–10 = intense [16]. Each panelist first independently assessed the typical size, appearance and uniform ripeness of fruits within each variety. They then randomly selected two fruits from each variety for palate evaluation. Samples remained unnamed until the completion of all evaluations to ensure objectivity. All assessments were conducted at 11 a.m. in individual sensory booths under white light [7].

### 2.3. E-Nose Measurement

Half of the 50 samples were randomly divided into 30 blocks for E-nose measurement, with each block analyzed in triplicate. The measurements were carried out using a commercially available portable E-nose (PEN3.5, Airsense Analytics GmbH, Schwerin, Germany). The sensor array of the PEN3.5 comprises ten metal oxide semiconductor chemical sensors (Table 2), which can operate at high temperatures, enabling the classification and identification of various volatile substances. When these sensors are exposed to volatile compounds, the ratio of the changed conductivity (G) to the initial conductivity (G0) (G/G0, referred to as relative conductivity or response value) varies depending on G. The concentration of the volatile substances leads to a deviation of G/G0 from 1 (either greater than or less than 1).

In this study, 10 g of flesh cubes from each sample were placed in a 300 mL beaker, which was then sealed with a sealing film. The beaker was maintained at 25 °C for 30 min in preparation for E-nose evaluation. The aroma measurement method was adapted from Afkari-Sayyah et al. [17] with slight modifications. Specifically, the E-nose sampling needle was inserted through the sealing film to extract the gas from the beaker for detection. The volatile gas was pumped over the E-nose sensors at a flow rate of 400 mL/min, and the E-nose analyses were recorded over a 60-s period (from 0 to 60 s). During the middle stage of data stabilization, 1–3 stable signals (response values) were observed. After each analysis, the sampling chamber was purged with a stream of dried air for 60 s to ensure cleanliness for subsequent measurements.

### 2.4. GC-MS Measurement

For quality control of the study, data quality control (QC) samples were equal mixes of the experimental samples and were used to balance the chromatography–mass spectra as well as to monitor the stability of the instrument and the reproducibility of the analytical samples under the same processing method. The repeatability of metabolite extraction and detection can be evaluated through overlapping display analysis of the total ion current chromatograms (TIC plots) derived from mass spectrometric analyses of various quality control (QC) samples.

The GC-MS method employed in this study was adapted with modifications from the protocols described by Zhang et al. [18]. Fruit volatiles were sampled using headspace solid-phase microextraction (HS-SPME) with a 65 μm polydimethylsiloxane-divinylbenzene (PDMS-DVB) fiber. For each sample, 5 g of fresh flesh (fresh weight) was ground into peach purée under liquid nitrogen prior to analysis. The samples were weighed into vials, which were then placed in a 30 °C water bath for 2 min. To ensure homogenization, 4 mL of saturated NaCl solution (as a matrix modifier) and 10 μL of 0.0819 μg·μL^−1^ 2-octanol (as an internal standard) were added to each vial. The vials were preincubated at 40 °C for 30 min under continuous agitation at 500 rpm, followed by volatile extraction at the same temperature and agitation speed for another 30 min. After extraction, the SPME fiber was desorbed in the GC injection port for 5 min in splitless mode.

The GC analysis was performed using a 7890A chromatograph (Agilent Technologies, Santa Clara, CA, USA) equipped with a DB-wax capillary column (30 m × 0.32 mm × 0.25 μm; J&W Scientific, Folsom, CA, USA), with helium as the carrier gas at a constant flow rate of 1.6 mL·min^−1^. The GC temperature program was set as follows: initial temperature of 34 °C held for 2 min, increased at 2 °C·min^−1^ to 60 °C, then ramped at 5 °C·min^−1^ to 220 °C and held at 220 °C for 2 min. The temperatures of the injection port, interface and MS source were set to 250 °C, 260 °C and 230 °C, respectively. Mass spectrometry was conducted using a 5975C mass spectrometer (Agilent Technologies) with an ionization potential of 70 eV, a scanning speed of 7 scans per second and electron ionization (EI) mode.

Volatile compounds were identified by comparing their electron ionization mass spectra and retention times with those in the NIST/EPA/NIH mass spectral library (NIST-08). Additionally, lactone volatiles were confirmed by comparison with authentic standards (Sigma, Burlington, MA, USA). The concentrations of volatiles were quantified using the peak area of the internal standard as a reference, based on the total ion chromatogram (TIC). The odor activity value (OAV) of each volatile compound was calculated as the ratio of its concentration to its odor threshold (odor thresholds were obtained from Cariou, 2016 [19]). Compounds with an OAV > 1 were considered to contribute significantly to the fruit’s odor profile.

### 2.5. Statistical Analysis

A range of software packages was employed for the analysis of electronic nose (E-nose) data, such as WinMuster (integrated in PEN3.5, Airsense Analytics GmbH, Schwerin, Germany), IBM SPSS Statistics 22.0 (IBM, Armonk, NY, USA) and Minitab 18 (Minitab, State College, PA, USA). Excel 2016 was used to sort and analyze the data, Metware (https://cloud.metware.cn/#/tools/tool-list, accessed on 10 March 2023, Wuhan, China.) was used to analyze the metabolome data, such as PCA, OPLS-DA, correlation, clustering heat map, KEGG enrichment analysis, and differential metabolite screening.

## 3. Results

### 3.1. Sensory Attribute

Through statistical description of sensory evaluators, there was no special flavor in “Tachibanawase”, but others had special fragrances. “Jin Xia Zao You Pan” and “Zi Jin Hong 3” had gardenia flavor, and passion fruit was also shown in “Zi Jin Hong 3”. There was a weak peach flavor but strong rust in “Xuan Cheng Tian Tao”. “Mangold” appeared moderate peach and honeydew flavor. There was a very complex flavor in “Bai Mi Pan Tao”, including moderate milk, violet and strong peach.

**Figure 1:**
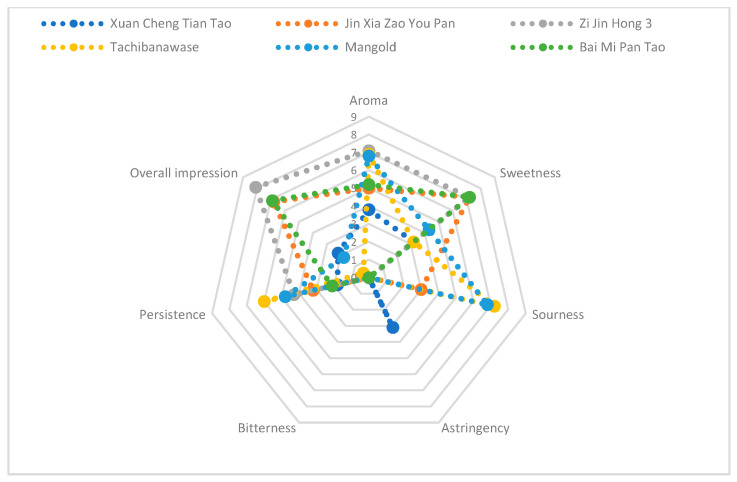
A radar map of sensory evaluations in six peaches and nectarines.

A radar map was made based on the score of aroma, sweetness, sourness, astringency, bitterness, persistence and overall impression description (Figure 1). “Zi Jin Hong 3” had high scores of aroma, sweetness, persistence and overall sensory quality, while low scores of sourness, astringency and bitterness description. “Jin Xia Zao You Pan”, “Zi Jin Hong 3” and “Bai Mi Pan Tao” had the same high sweetness scores, and the aroma score was 7.1 in “Zi Jin Hong 3”. The persistence evaluation of “Tachibanawase” and “Mangold” ranked first and second, higher than other varieties. The bitterness of six varieties in the radar map was not obvious, but “Xuan Cheng Tian Tao” had some astringency. The overall impression scores were higher in “Bai Mi Pan Tao”, “Zi Jin Hong 3” and “Jin Xia Zao You Pan”, while the lowest was in “Tachibanawase”. The results clearly demonstrated that “Tachibanawase” exhibited poor sensory quality, whereas “Bai Mi Pan Tao”, “Zi Jin Hong 3” and “Jin Xia Zao You Pan” achieved the highest scores for superior sensory quality (Table 3).

### 3.2. E-Nose Results

**Figure 2:**
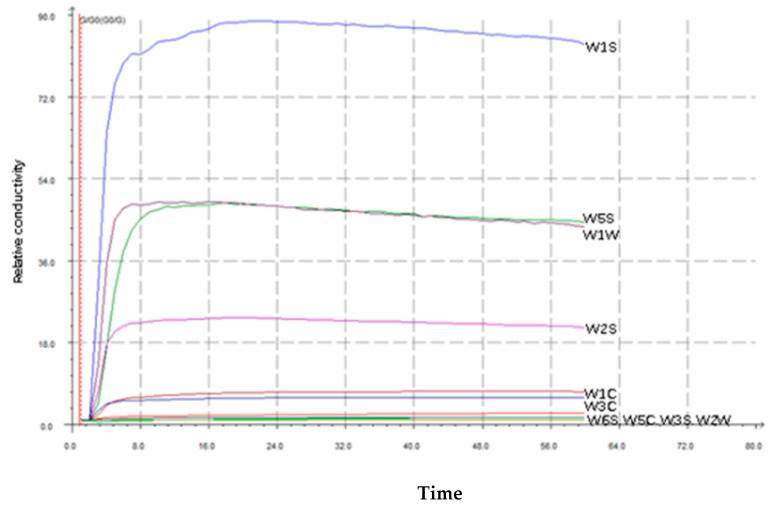
Bai Mi Pan Tao response values to different sensors of an electronic nose.

In Figure 2, the response values of peach fruit aroma to different sensors of electronic nose were displayed by Bai Mi Pan Tao. The response values of each sensor reached its maximum value around 24 s. Therefore, a stable period was 35 s to 37 s for subsequent test analysis. Ten sensors were W1S (methane) with the largest response value, and then W5S (nitrogen oxides), W1W (hydrogen sulfide), W6S (hydrogen), W5C (alkane), W3S (aromatic alkane) and W2W (aromatic components and organic sulfides).

**Figure 3:**
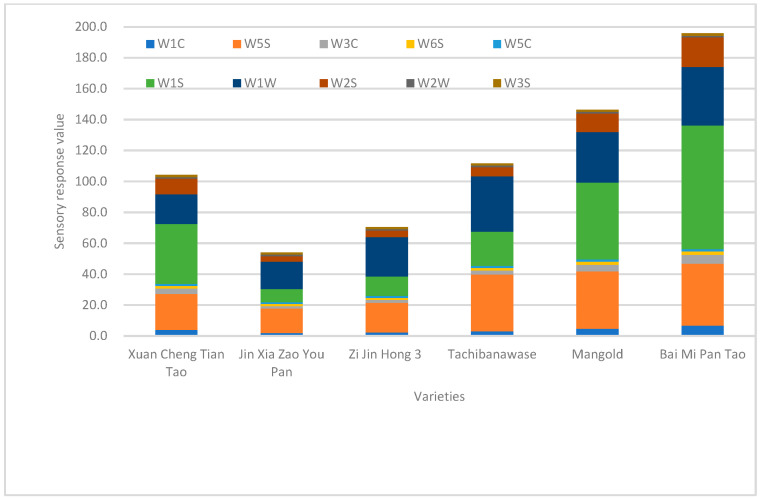
Response values to different sensors of an electronic nose in 6 peach varieties.

In Figure 3, the total sensory response values ranged from 54.04 (Jin Xia Zao You Pan) to 195.7 (Bai Mi Pan Tao). W1S, W1W and W5S were high values in all cultivars. The values in “Bai Mi Pan Tao” were 79.89 of W1S, 40.16 of W5S and 37.83 of W1W, while “Jin Xia Zao You Pan” had 8.58 in W1S, 15.82 in W5S and 17.80 in W1W. W5C, W2W and W3S had low values around 1 in all cultivars. Values of W3C and W2S in “Mangold” and “Bai Mi Pan Tao” were higher than other cultivars.

#### Principal Component Analysis of Population Sample

**Figure 4:**
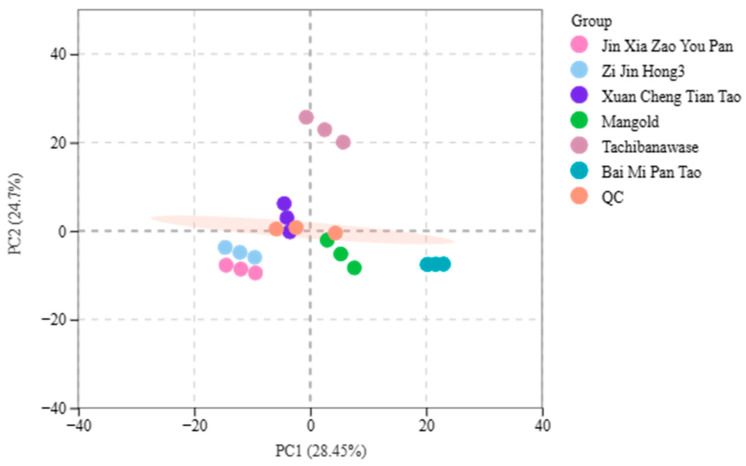
PCA analysis of samples.

Principal component analysis (PCA) of all samples—including quality control (QC) samples—allows for a preliminary grasp of the overall metabolite variations between different sample groups and the variability among samples within the same group. As shown in Figure 4, the contribution rates of the first and second principal components were 28.45% and 24.7%, respectively. The QC samples clustered near the “0” point, which confirms the stability and reliability of the test method and instrument used.

In terms of the sample distribution, “Tachibanawase” exhibited positive scores on both the first and second principal components. In contrast, “Zi Jin Hong 3” and “Jin Xia Zao You Pan” had negative scores on both components. “Mangold” and “Bai Mi Pan Tao” showed positive scores on the first principal component but negative scores on the second. “Xuan Cheng Tian Tao” displayed the opposite pattern, with negative scores on the first principal component and positive scores on the second. Overall, the six peach varieties showed a clear tendency to separate from one another, with little variability observed within each group.

### 3.3. Category Analysis of Metabolite Composition

**Figure 5:**
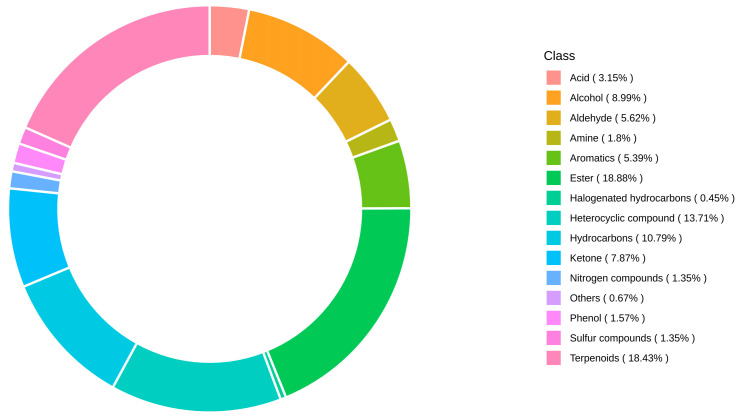
Metabolite composition category ring chart.

Totally, 446 metabolites were detected based on the GC-MS, including 86 esters, 82 terpenes, 61 heterocyclic compounds, 48 hydrocarbons, 40 alcohols, 35 ketones, 25 aldehydes, 24 aromatics, 14 acids, 8 amines, 7 phenols, 6 nitrogen compounds, 6 sulfides, 2 halogens and 3 other metabolites. The proportion of esters in metabolites was 18.88%. Terpenoids, heterocyclic compounds, hydrocarbons, alcohols, aldehydes, aromatics, acids, amines, phenolic nitrogen compounds and sulfides were 18.43%, 13.71%, 10.79%, 8.99%, 7.87%, 5.62%, 5.39%, 3.15%, 1.8%, 1.57%, 1.35% and 1.35%, respectively. Other metabolites and halogenates accounted for less than 1%, 0.67% and 0.45%, respectively. Esters and terpenes were the main volatile substance types of peach fruit aroma, accounting for 37.31%, as shown in Figure 5.

### 3.4. Metabolite Expression Analysis

**Figure 6:**
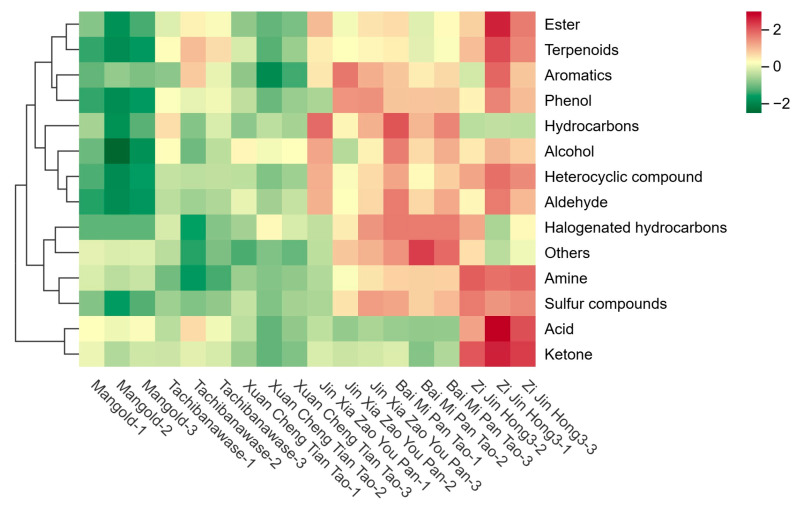
The horizontal coordinate of six peaches.

In Figure 6, the horizontal coordinate represented metabolites, and the vertical coordinate represented different samples. The color changed from red to green according to the level of expression. As the hues lean towards crimson, they signify elevated levels of the metabolite’s expression; while verdant shades bespeak a downtrend in the metabolite’s expression levels. The expression levels of various metabolites in “Mangold” and “Bai Mi Pan Tao” were lower, compared with other four cultivars.

**Figure 7:**
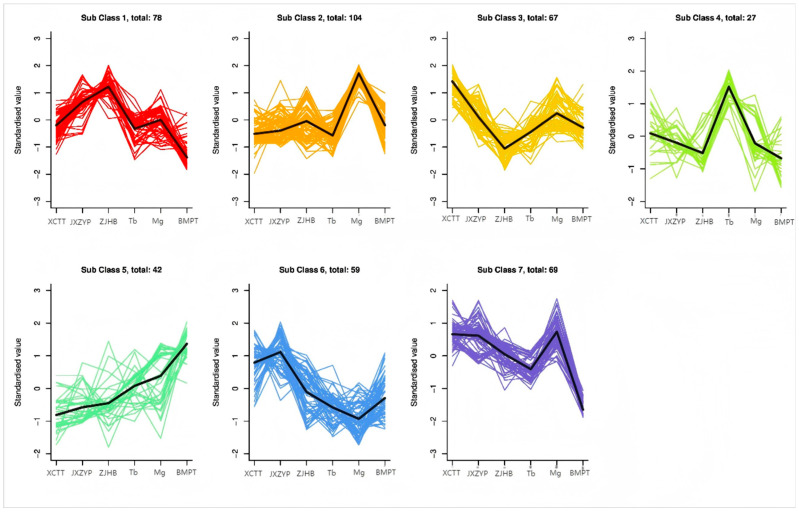
K-means clustering of 6 peaches.

The change trend of relative metabolite content, data standardization and clustering of different metabolite content were conducted in peaches. The results of K-means clustering (Figure 7) showed that the 446 metabolites in six peaches could be divided into seven categories, including 78, 104, 67, 27, 42, 59 and 69 metabolites in the seven categories. In cluster I, II and VII, the content of metabolites in “Mangold” was significantly lower than that in others, and these three clusters contained 251 metabolites.

OPLS-DA can maximise the differences between groups, which was conducive to finding differential metabolites (Table 4). As shown in Table 4, the Q^2^ values for all treatments exceeded 0.9, demonstrating that all models are stable and reliable, and thus suitable for further screening of differential metabolites.

**Figure 8:**
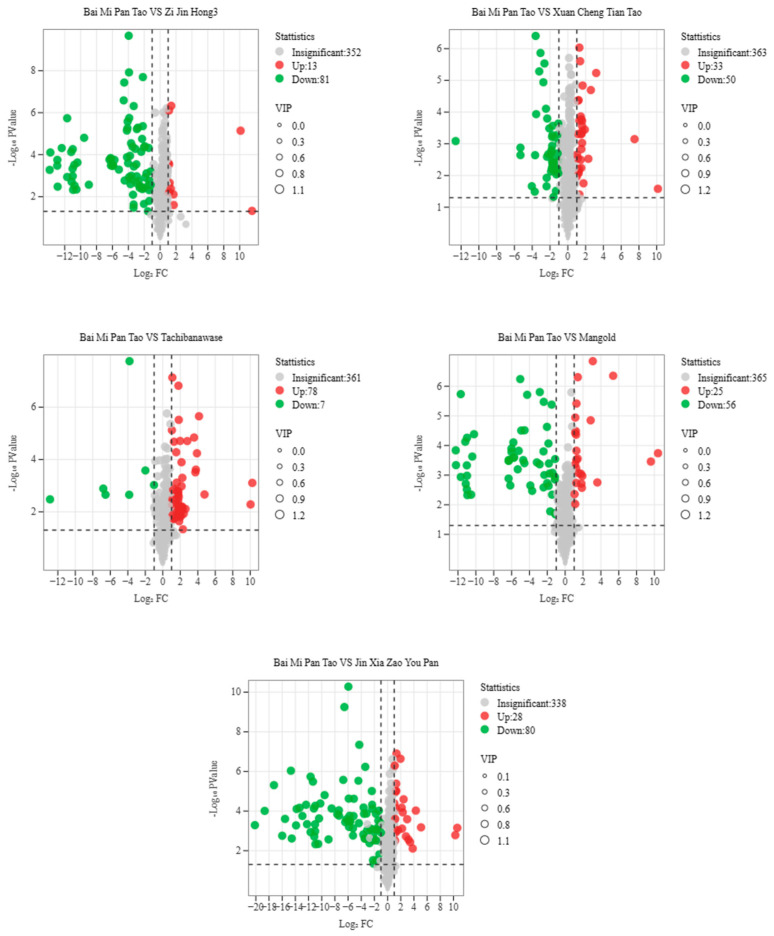
Volcano plot of differential metabolites. Note: Each dot in the graph stands for a metabolite: green dots denote downregulated differential metabolites, red dots represent upregulated differential metabolites, and gray dots indicate metabolites that were detected but showed no significant differences. The abscissa corresponds to the logarithmic value (log_2_FC) of a metabolite’s relative content in the two sample groups; the greater the absolute value of the abscissa, the more pronounced the difference in the metabolite’s relative content between the two groups.

Combined with OPLS-DA analysis, KEGG enrichment statistics were performed for differential metabolites. Based on the results of differential analysis, metabolites with VIP ≥ 1 and T-test (*p* < 0.05) were screened as significant differential metabolites in this study. The results (Table 5 and Figure 8) showed that the total number of differential metabolites of “Bai Mi Pan Tao” vs. “Xuan Cheng Tian Tao”, “Bai Mi Pan Tao” vs. “Jin Xia Zao You Pan”, “Bai Mi Pan Tao” vs. “Zi Jin Hong 3”, “Bai Mi Pan Tao” vs. “Tachiwanawase” and “Bai Mi Pan Tao” vs. “Mangold” were 83, 108, 94, 85 and 81, with 33, 28, 13, 78, 25 up-regulated metabolites and 50, 80, 81, 7, 56 down-regulated differential metabolites, respectively. Differential metabolites in four groups, besides “Bai Mi Pan Tao” vs. “Tachiwanawase”, were dominated by the down-regulated mode.

**Figure 9:**
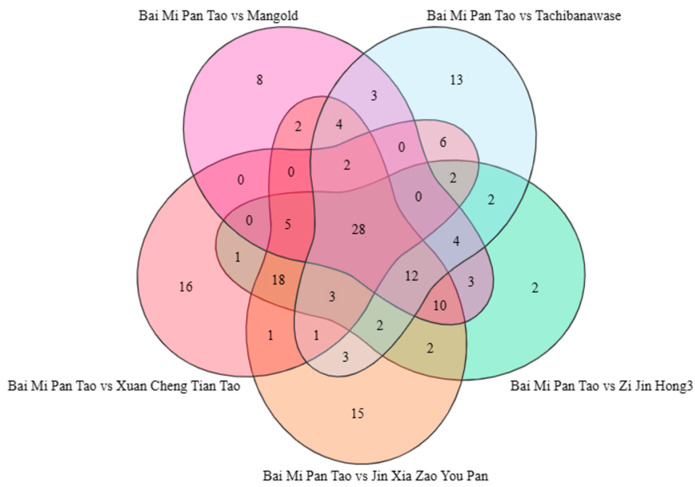
Venn diagram of metabolites. Note: Each circle in the figure corresponds to a comparison group. The numbers within the circles—including those in the overlapping regions—indicate the count of differential metabolites shared between the relevant comparison groups, while the numbers in the non-overlapping regions represent the differential metabolites unique to a specific comparison group.

The Wayne diagram showed the relationship between groups of different metabolites (Figure 9). A total of 28 metabolites were found in all groups, including two alcohols, six aromatics, one phenol, one aldehyde, two acids, two terpenoids, one hydrocarbon, four ketones, two heterocycles and seven esters. There were 16 specific metabolites in Bai Mi Pan Tao vs. Xuan Cheng Tian Tao (aromatic compounds: Benzene, 1,2,4-trimethyl- and 2-Hydroxyfluorene), 15 specific metabolites in Bai Mi Pan Tao vs. Jin Xia Zao You Pan (aromatic compounds: BenzAldehyde, 4-methyl-, Benzenemethanol, 4-methyl-, Benzene, (1-methoxypropyl)- and Benzenemethanethiol), 2 specific metabolites in Bai Mi Pan Tao vs. Zi Jin Hong 3 (aromatic compounds: (R)-(+)-1-(p-Tolyl) ethylAmine), 13 specific metabolites in Bai Mi Pan Tao vs. Tachiwanawase (aromatic compounds: Azulene and Acetophenone, 4′-hydroxy-), and 8 specific metabolites in Bai Mi Pan Tao vs. Mangold. Details of the specific metabolites between the comparison groups are shown in Table 6.

**Figure 10:**
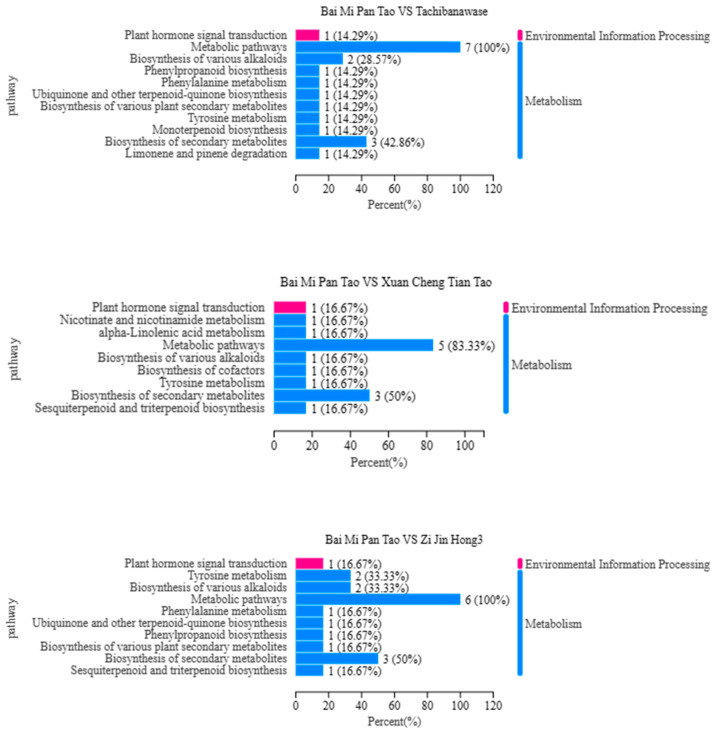

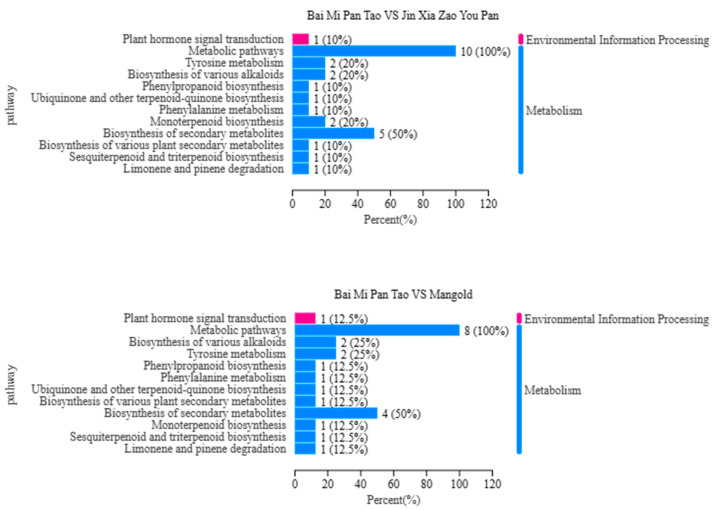
Differential metabolite KEGG enrichment analysis.

In Figure 10, the Rich Factor is defined as the ratio of the number of differential metabolites to the total number of metabolites, while the percentages represent the proportion of metabolites enriched in the corresponding pathways. In the significant enrichment analysis, through pathway annotation of differential metabolites, it was found that the KEGG (Kyoto Encyclopedia of Genes and Genomes) metabolic pathways of differential metabolites in each comparison group mainly included metabolism and environmental information processing. Among these, the only pathway under environmental information processing was plant hormone signal transduction.

Specifically, the metabolic pathways of differential metabolites in the “Bai Mi Pan Tao vs. Xuan Cheng Tian Tao” group mainly included nicotinate and nicotinamide metabolism, α-linolenic acid metabolism, metabolic pathways, biosynthesis of various alkaloids, biosynthesis of cofactors, tyrosine metabolism, biosynthesis of secondary metabolites, and sesquiterpenoid and triterpenoid biosynthesis.

For the “Bai Mi Pan Tao vs. Jin Xia Zao You Pan” group, the relevant metabolic pathways primarily encompassed metabolic pathways, tyrosine metabolism, biosynthesis of various alkaloids, phenylpropanoid biosynthesis, ubiquinone and other terpenoid-quinone biosynthesis, phenylalanine metabolism, monoterpenoid biosynthesis and biosynthesis of secondary metabolites.

In the “Bai Mi Pan Tao vs. Zi Jin Hong 3” group, the metabolic pathways of differential metabolites mainly included tyrosine metabolism, biosynthesis of various alkaloids, phenylalanine metabolism, ubiquinone and other terpenoid-quinone biosynthesis, phenylpropanoid biosynthesis, biosynthesis of various plant secondary metabolites, and biosynthesis of secondary metabolites.

For the “Bai Mi Pan Tao vs. Tachiwanawase” group, the metabolic pathways mainly involved metabolic pathways, biosynthesis of various alkaloids, phenylpropanoid biosynthesis, phenylalanine metabolism, ubiquinone and other terpenoid-quinone biosynthesis, biosynthesis of various plant secondary metabolites, tyrosine metabolism and monoterpenoid biosynthesis.

Lastly, in the “Bai Mi Pan Tao vs. Mangold” group, the metabolic pathways of differential metabolites mainly included metabolic pathways, biosynthesis of various alkaloids, tyrosine metabolism, phenylpropanoid biosynthesis, phenylalanine metabolism, ubiquinone and other terpenoid-quinone biosynthesis, biosynthesis of various plant secondary metabolites and biosynthesis of secondary metabolites.

## 4. Discussion

Sensory evaluation is an important method for assessing peach fruit quality. In this study, the aroma profiles of six varieties were evaluated based on color, aroma intensity, taste, aftertaste and overall impression. Building on our previous research, six representative peach samples—”Xuan Cheng Tian Tao”, “Jin Xia Zao You Pan”, “Zi Jin Hong 3”, “Tachibanawase”, “Mangold” and “Bai Mi Pan Tao”—were selected from a collection of 356 germplasm accessions. Sensory evaluation and electronic nose analysis revealed that all six peach varieties possess a distinct peach aroma complemented by floral and milky notes [7]. Notably, the W5S and W1W sensors showed significantly higher response values than other sensors. These findings align with metabolomic results indicating that esters and lactones constitute the primary aroma components in mature peaches. The characteristic fruity and sweet fragrance perceived in peaches originates mainly from esters, while lactones impart the distinctive peachy aroma. Each lactone compound contributes specific odor attributes, including fruity (peach/coconut), sweet, creamy, caramel, floral and herbal notes [3,20].

Previous studies have revealed that γ-lactone and delta-lactone play a significant role in the development of the milk flavor in peach [6,21]. Delta-decalactone and delta-dodecalactone are the characteristic components of the cream flavor, especially delta-decalactone has a strong and long-lasting sweet cream aroma in peaches, so it can be inferred that the milk flavor of Bai Mi Pan Tao is due to delta-decalactone. Delta-decanolactone also gives sweet apples, apricots, mangoes and other fruits flavor in fruit [21,22]. In this study, six peaches and nectarines were used as materials, and a total of 446 metabolites were detected by GC-MS metabolomics analysis. A total of 28 metabolites were found in six peaches and nectarines, including two alcohols, six aromatics, one phenol, one aldehyde, two acids, two terpenes, one hydrocarbon, four ketones, two heterocycles and seven esters. The seven esters were 1-octene-3-butanate,6, 6-dimethyl-bicyclic [3.1.1] hept-2-ene-2-carboxylate, 2-methylpropionate, 1-methyl-4 -(1-methylvinyl) cyclohexyl acetate, (4-methyl-1-propane-2-yl-3-bicyclic [3.1.0] hexyl) acetate and isobutyl benzoate Cis-6-nonene-1-ol acetate, indicating that the special aroma perceived by people in Xuan Cheng Tian Tao, Jin Xia Zao You Pan, Zi Jin Hong 3, Tachibanawase, Mangold and Bai Mi Pan Tao.

In addition to esters, terpenoids are also the main components of peach fruit aroma [3,23]. In this study, the terpenoids of 28 common substances detected were mainly 1-Oxaspiro [4.5]dec-6-ene, 2,6,10,10-tetramethyl- and dl-Camphoroquinone. According to the results of sensory analysis of fruit aroma types, the aroma type of “Xuan Cheng Tian Tao” is wood, which may be due to the existence of 1-Oxaspiro [4.5]dec-6-ene, 2,6,10,10-tetramethyl-. According to the sensory results, the aroma type of “Jin Xia Zao You Pan” is floral, which may be due to the presence of BenzAldehyde. The aroma type of “Zi Jin Hong 3” is fruity and floral, which may be due to the fruity esters, Butanoic acid, 1-ethenylhexyl ester, Methyl 6,6-dimethylbicyclo [3.1.1]hept-2-ene-2-carboxylate, Propanoic acid, 2-methyl-, phenylmethyl ester, Cyclohexanol, 1-methyl-4-(1-methylethenyl)-, acetate, Iso-3-thujyl acetate, Benzoic acid, 2-methylpropyl ester and 6-Nonen-1-ol, acetate, (Z)-. Mangold has a fruity flavor related to the ester volatile substance “Acetic acid, heptyl ester”. “Tachiwanawase” has an unpleasant smell, due to the volatile compounds, Di-epi-.alpha.-cedrene-(I), 2,3-Dehydro-1,8-cineole, (1R,3aS,4aS,8aS)-1,4,4,6-Tetramethyl-1,2,3,3a,4,4a,7,8-octahydrocyclopenta [1,4]cyclobuta [1,2]benzene, Panaxene, Cyclohexene, 3-(1,5-dimethyl-4-hexenyl)-6-methylene-, [S-(R*,S*)]- and 1,7-Octadiene, 2-methyl-6-methylene-.

The sensory evaluation, electronic nose analysis and metabolomics in this study lay in quality differences through multi-level associations of “sensory phenotype—volatile compounds—metabolic mechanisms,” which cannot be limited to peaches. Thus, the sample processing protocols (e.g., optimized HS-SPME-GC-MS parameters) and data analysis methods (e.g., PCA/OPLS-DA for screening differential metabolites) established in this study can be applied to quality analysis of other agricultural products with only minor adjustments based on the volatile characteristics of the target fruit (e.g., modifying electronic nose sensor combinations or optimizing GC-MS temperature programs) [24].

## 5. Conclusions

The integration of sensory evaluation, E-nose analysis and metabolomics provided a comprehensive understanding of the aroma differences among peach varieties. The findings highlight the systematic identification of characteristic flavor compounds and their metabolic basis in peach germplasm resources, which can provide molecular markers for breeding high-quality cultivars with charming flavor. In agricultural product processing, it can optimize processing techniques (e.g., fermentation conditions and storage methods) based on characteristic metabolites (such as esters and terpenes in this study) to reduce flavor loss. Additionally, due to the characteristics of peach fruit—including vigorous postharvest physiological metabolism and poor storage performance—the study offers a theoretical foundation for raw material selection and process optimization in the deep-processing industry of peach fruits, such as wine brewing and dehydration for dried products, in future research. Future research can further apply this system to cross-crop quality comparisons, providing more comprehensive technical support for “flavor standardization” and “high-quality cultivar breeding” in pomology.

## Figures and Tables

**Table 1:** Peach and nectarine varieties used in this study.

No.	Name	Harvest Date	Species or Variety	Country of Origin	Type	Color of the Flesh
1.	Xuan Cheng Tian Tao (XCTT)	7 June	*P. persica* (L.) Batsch	China	Peach	Red
2.	Jin Xia Zao You Pan (JXZYP)	15 June	*P. persica* var. nectarina (Ait.) Maxim	China	Nectarine	Yellow
3.	Zi Jin Hong 3 (ZJH3)	23 June	*P. persica* var. nectarina (Ait.) Maxim	China	Nectarine	Yellow
4.	Tachibanawase (Tb)	1 July	*P. persica* (L.) Batsch	Japan	Peach	White
5.	Mangold (Mg)	5 July	*P. persica* (L.) Batsch	USA	Peach	Yellow
6.	Bai Mi Pan Tao (BMPT)	2 August	*P. persica* var. compressa Bean.	China	Peach	White

**Table 2:** Electronic nose sensor array (PEN 3.5) using the portable E-nose (Air-sense Analytics GmbH, Germany).

Sensor Name	Sensor Sensitives
W1C	Sensitive to aromatic benzene
W3C	Sensitive to ammonia and aromatic compounds
W5C	Sensitive to nitrogen oxides
W1S	Sensitive to short-chain alkanes such as methane
W2S	Sensitive to alcohols, ethers, aldehydes and ketones
W3S	Sensitive to long-chain alkanes
W5S	Sensitive to hydrocarbons and aromatic compounds
W6S	Sensitive to hydrogen
W1W	Sensitive to terpenes and organosulfur compounds
W2W	Sensitive to aromatic compounds and sulphur and chlorine compounds

**Table 3:** Scores of sensory evaluations in six peaches and nectarines.

Name	Aroma	Sweetness	Sourceness	Astrigency	Bitterness	Persistnece	Overall Impression	Special Flavour
Xuan Cheng Tian Tao (XCTT)	3.8	3.2	0	3.1	0	1.8	2.2	Peach
Jin Xia Zao You Pan (JXZYP)	5	7.2	3	0	0	3.2	6.8	Gardenia
Zi Jin Hong 3 (ZJH3)	7.1	7.2	0	0	0	4.3	6.9	Gardenia; passion fruit
Tachibanawase (Tb)	6.9	3.2	7.2	0	0	6	0.4	
Mangold (Mg)	6.8	4.3	6.8	0	0	4.8	1.8	Peach; honeydew
Bai Mi Pan Tao (BMPT)	5.2	7.2	0	0	0	2.1	8.1	Peach; moderate milk; violet

**Table 4:** OPLS-DA analysis results for all groups.

Group	R2X	R2Y	Q2
Bai Mi Pan Tao vs. Xuan Cheng Tian Tao	0.888	1.000	0.998
Bai Mi Pan Tao vs. Jin Xia Zao You Pan	0.933	1.000	0.997
Bai Mi Pan Tao vs. Zi Jin Hong 3	0.922	1.000	0.997
Bai Mi Pan Tao vs. Tachiwanawase	0.899	1.000	0.995
Bai Mi Pan Tao vs. Mangold	0.856	1.000	0.995

**Table 5:** The total number of differential metabolites.

Group Name	All Sig Diff	Down Regulated	Up Regulated
Bai Mi Pan Tao vs. Xuan Cheng Tian Tao	83	50	33
Bai Mi Pan Tao vs. Jin Xia Zao You Pan	108	80	28
Bai Mi Pan Tao vs. Zi Jin Hong 3	94	81	13
Bai Mi Pan Tao vs. Tachiwanawase	85	7	78
Bai Mi Pan Tao vs. Mangold	81	56	25

**Table 6:** Differential metabolites specific to each comparison group.

“Bai Mi Pan Tao” vs. “Xuan Cheng Tian Tao”	“Bai Mi Pan Tao” vs. “Jin Xia Zao You Pan”	“Bai Mi Pan Tao” vs. “Zi Jin Hong 3”	“Bai Mi Pan Tao” vs. “Tachiwanawase”	“Bai Mi Pan Tao” vs. “Mangold”
Octanal	2,7-Octadien-4-ol, 2-methyl-6-methylene-, (S)-	(R)-(+)-1-(p-Tolyl) ethylamine	Isomaltol	1-Octanol
Isobutyl isovalerate	Ethanone, 1-(2-thienyl)-	2-Furanmethanol, tetrahydro-α, α,5-trimethyl-5-(4-methyl-3-cyclohexen-1-yl)-, [2S-[2α,5β (R*)]]-	Azulene	Butanoic acid, 2-methyl-, 3-methylbutyl ester
Acetic acid, hexyl ester	Hotrienol		2-Cyclopentylethanol	2-Nonanol
3-Hexene, 1-(1-methoxyethoxy)-, (Z)-	Benzaldehyde, 4-methyl-		Di-epi-.α.-cedrene-(I)	Acetic acid, heptyl ester
Acetic Acid, (acetyloxy)-	7-Octen-4-ol, 2-methyl-6-methylene-, (S)-		2,3-Dehydro-1,8-cineole	Undecane
3-Hexen-1-ol, acetate, (Z)-	Benzenemethanol, 4-methyl-		cis-2,6-Dimethyl-2,6-octadiene	2-Isopropyl-5-methylhex-2-enal
Isospathulenol	Lilac Alcohol C		Acetophenone, 4′-hydroxy-	Pyrazine, 2,3-dimethyl-5-(1-methylpropyl)-
2,6,11,15-Tetramethyl-hexadeca-2,6,8,10,14-pentaene	Benzene, (1-methoxypropyl)-		2,4-Heptadien-1-ol, (E,E)-	2,4-Dimethyl-2-oxazoline-4-methanol
N,N-Dimethylhexanamide	Benzyl angelate		(1R,3aS,4aS,8aS)-1,4,4,6-Tetramethyl-1,2,3,3a,4,4a,7,8-octahydrocyclopenta [1,4]cyclobuta [1,2]benzene	
Benzene, 1,2,4-trimethyl-	L-α-Terpineol		Panaxene	
2-Hydroxyfluorene	Butanoic acid, 4-hexenyl ester, (Z)-		Cyclohexene, 3-(1,5-dimethyl-4-hexenyl)-6-methylene-, [S-(R*,S*)]-	
5-Isoxazolecarboxylic acid, 4,5-dihydro-5-methyl-, methyl ester, (R)-	Benzenemethanethiol		1,7-Octadiene, 2-methyl-6-methylene-	
trans-3-Methyl-4-octanolide	1-Hepten-6-one, 2-methyl-		2,6-Dodecadien-1-al	
Thiazol-2-Amine, N-(4-dimethylaminobenzyl)-	Hexanoic acid, propyl ester			
Niacinamide	Benzoic acid, 1-methylethyl ester			
Phenanthrene, 7-ethenyl-1,2,3,4,4a,4b,5,6,7,9,10,10a-dodecahydro-1,1,4a,7-tetramethyl-, [4aS-(4a.α.,4b.β,7 β,10a.β)]-				

## Data Availability

The original contributions presented in this study are included in the article. Further inquiries can be directed to the corresponding authors.
